# Structural characterization and anti-inflammatory activity of a novel polysaccharide PKP2-1 from *Polygonatum kingianum*

**DOI:** 10.3389/fnut.2023.1156798

**Published:** 2023-03-27

**Authors:** Zhen Wang, Hui Liu, Ranze Fu, Jinmei Ou, Bin Wang

**Affiliations:** Key Laboratory of Xin’an Medicine of the Ministry of Education, Anhui University of Chinese Medicine, Hefei, China

**Keywords:** polysaccharide, *P. kingianum*, structural elucidation, anti-inflammatory activity, MH7A cell

## Abstract

**Introduction:**

This study aimed to investigate the structure characterization and antiinflammatory activity of a novel polysaccharide, PKP2-1, from the rhizomes of *Polygonatum kingianum* Coll. and Hemsl.

**Methods:**

We isolated a novel polysaccharide, PKP2-1, from the rhizomes of *Polygonatum kingianum* Coll. and Hemsl. for the first time, which was then successively purified through hot-water extraction, 80% alcohol precipitation, anion exchange and gel permeation chromatography. The *in vitro* anti-inflammatory activity of PKP2-1 in MH7A cells was assessed using a CCK-8 kit assay.

**Results:**

Monosaccharide composition assay revealed that PKP2-1 was mainly composed of glucose, galactose, mannose, and glucuronic acid at an approximate molar ratio of 6:2:2:1. It had a molecular weight of approximately 17.34 kDa. Structural investigation revealed that the backbone of PKP2-1 consisted of (→2, 3)-α-D-Galp(4→, →2)-α-D-Manp(3→, →2)-β-D-Glcp(4→) and α-D-Glcp(3→) residues with side chains (→2)-β-D-Glcp(4→, →1)-α-D-Galp(4→) and α-D-Glcp(3→) branches located at O-3 position of (→2, 3)-α-D-Galp(4→). The *in vitro* anti-inflammatory activity of PKP2-1 in MH7A cells revealed that PKP2-1 could reduce the expression of IL-11β and IL-6, increase the expression of IL-10 and induce apoptosis of synovial fibroblasts.

**Conclusion:**

The PKP2-1 could inhibit MH7A cell growth and potentially be exploited as an anti-inflammatory agent.

## 1. Introduction

Inflammation often occurs during the dysfunction of the body’s immune system and tissue damage. It is accompanied by the release of related mediators, including tumor necrosis factor-alpha (TNF-α), interleukin (IL-1β, IL-6, IL-10, etc.), and so on (1). Although some synthetic anti-inflammatory drugs can positively reduce inflammation (2), long-term intake could cause adverse events such as liver toxicity. In addition to resveratrol, curcumin, capsaicin, colchicine, quercetin, epigallocatechin-3-gallate (3) and others, plant-derived and low-toxicity anti-inflammatory compounds such as polysaccharides have also contributed significantly in the development of traditional Chinese medicine (TCM).

A polysaccharide is a carbohydrate bonded by linear or branched chain glycosidic bonds, formed by condensation reaction among more than ten monosaccharides. In nature, there are diverse polysaccharides, such as starch, cellulose and animal cell wall (4). Notably, many TCMs such as *Polygonati* (5), *Astragalus* (6), *Angelica* (7), *Achyranthes* (8), and *Poria* (9) contain various polysaccharide components. TCMs polysaccharides have versatile bioactivities such as anti-inflammatory, immunomodulatory, anti-oxidation and other effects (10, 11), indicating that unique TCMs polysaccharide structure could be responsible for its biological efficacy. However, since TCMs polysaccharides feature a diversity of monosaccharide species, special selectivity of connection mode and non-homogeneous molecular size, structure elucidation for TCMs polysaccharides is difficult and extremely challenging.

As is well-known, *Polygonati* Rhizoma, known as “Huangjing,” was first documented in the well-known book Mingyi Bielu, written in the Chinese Han Dynasty (12). The dried rhizomes of *P. kingianum* Coll. et Hemsl. (13), *P. sibiricum* Red. (14), and *P. cyrtonema* Hua (15) are defined as *Polygonati* Rhizoma sources and are legally recorded in the Chinese Pharmacopoeia. Due to its double efficacy in treating diseases and satisfying hunger, it is exploited not only as a tonic but also as nourishing food. It is also known as a homology of medicine and food in China (16, 17). *Polygonati* Rhizoma mainly contains polysaccharide and steroidal saponin components. Of these, polysaccharides are the main pharmacodynamic portions and are defined as the quality marker (Q-Marker) (18, 19).

Notably, polysaccharides from *Polygonatum* species are reported to possess anti-inflammatory, immunoregulatory, anti-osteoporosis, and antidiabetic effects (15, 20, 21), indicating that their bioactivity could be significantly associated with their structure. Recently, Yan et al. reported a novel P. *sibiricum* polysaccharide (PSP) consisting of (→2)-β-D-Galp-(1→) had remarkable osteogenic activity (20). Furthermore, two P. *cyrtonema* polysaccharides (PCP) were reported to contain β-D-Fruf and α-D-Glcp, which possessed significant immunomodulatory activities and hepatoprotective effects, respectively (15). More recently, Duan et al. developed a (1, 2)-linked β-D-Glucan obtained from crude P. *kingianum* polysaccharides (PKP) and found that it had important hypoglycemic efficacy (21). However, the anti-inflammatory potential of P. *kingianum* polysaccharides (PKP) has rarely been studied. In our previous studies, the polysaccharide portion purified from the *Polygonati* species was mainly composed of glucose, glucuronic acid, galactose, and galacturonic acid (22, 23).

Herein, we purified PKP from P. *kingianum*. According to the analysis for monosaccharide composition and molar ratio, combined with the result of methylation analysis, high-performance gel permeation chromatography (HPGPC), 2D nuclear magnetic resonance (NMR) and FT-IR, we assessed the detailed structure and *in vitro* anti-inflammatory activities of PKP-1 using an MH7A cell model. These findings provide a practical perspective for exploiting and applying *Polygonati* Rhizoma polysaccharides as an antiphlogistic drug.

## 2. Materials and methods

### 2.1. Materials

The dried rhizomes of *P. kingianum* were obtained from Bozhou Tongdefu pharmacy Co., Ltd. (Bozhou, China) and identified by Dr. Jinmei Ou, Anhui University of Chinese Medicine. A voucher specimen (2021–0712) was deposited at the Key Laboratory of Xin’an Medicine of the Ministry of Education, Anhui University of Chinese Medicine.

### 2.2. Reagents and chemicals

Sephadex G-200 and DEAE cellulose-52 were purchased from Kuer Co., Ltd. (Hefei, China). Trifluoroacetic acid (TFA), hydrogen peroxide and 1,1-diphenyl-2-picrylhydrazyl (DPPH) were obtained from Shanghai Macklin Biochemical Co., Ltd. (Shanghai, China). The National Institute for Food and Drug Control (Beijing, China) provided dextrans of a series of molecular weights (Mw: 1, 5, 12, 50, and 150 kDa). Sigma-Aldrich provided the monosaccharide standards for rhamnose (Rha), arabinose (Ara), fructose (Fru), galactose (Gal), glucose (Glc), xylose (Xyl), mannose (Man), galacturonic acid (GalA), and glucuronic acid (GlcA). Fetal bovine serum (FBS), Phosphate Buffered Saline (PBS), Dulbecco’s modified Eagle medium (DMEM), Cell Counting Kit-8, 4% Paraformaldehyde solution, Trypsin-EDTA and Crystal Violet Staining Solution, human TNF-α, Annexin V-FITC Apoptosis Detection Kit, Cell Cycle Staining kits, Human interleukin-1β ELLSA Kit (IL-1β), Human interleukin-10 ELLSA Kit (IL-10) and Human interleukin-6 ELLSA Kit (IL-6) were purchased from different manufacturers.

### 2.3. PKP2-1 preparation

The rhizomes of P. *kingianum* were processed using boiling water (1:4 w/v) for 30 min, and then 80% ethanol solution was used to precipitate the crude polysaccharide. The Sevag method, using CHCl_3_ and n-BuOH as reagent (4:1, v/v), was implemented to remove impure proteins (24). Finally, crude PKPs were obtained. Combined anion-exchange and gel-filtration chromatography and monitored using the colorimetric method (Phenol-sulfuric acid method) (25, 26), the crude PKPs were further purified to generate the desired homogeneous polysaccharide, defined as PKP2-1. Briefly, a crude PKPs sample weighing approximately 800 mg was dissolved into 80 mL of ultra-pure water. The prepared PKP solution was transferred into a DEAE-cellulose column (3.5 × 30 cm) and eluted using ultra-pure water and 0.05 M sodium chloride solution to yield PKP1 and PKP2, respectively. Further, the PKP2 portion was fractionated using a Sephadex G-200 column (1.6 × 40 cm) with ultra-pure water at a flow rate of 0.2 mL/min. The eluting peak was collected, concentrated and lyophilized under a vacuum to yield a highly purified polysaccharide (PKP2-1).

### 2.4. Structural elucidation

#### 2.4.1. Total sugar determination

The total sugar content was determined with glucose as the reference using the anthrone-sulfuric acid method with a slight modification (27). Briefly, the 33 mg anhydrous glucose was dissolved into 100 mL of deionized water to obtain a 0.33 mg/mL glucose standard solution. An indicated amount of standard solution, 0, 0.2, 0.4, 0.6, 0.8, and 1.0 mL, were, respectively taken into a centrifuge tube and diluted to 1.0 mL using ultra-pure water. Subsequently, 4 mL of 0.2% anthrone-sulfuric acid solution was slowly added. The reaction mixture was held in a boiling water bath for 20 min. The absorbance was measured under a wavelength of 582 nm, and the corresponding data were analyzed to build standard equation I, *y* = 7.8508 × + 0.295, *R*^2^ = 0.9993. PKP2-1 was dissolved in ultra-pure water to prepare a 0.5 mg/mL polysaccharide solution. After moderate dilution, the PKP2-1 analytes were mixed with an anthrone-sulfuric acid solution. Finally, the absorbance of PKP2-1 analytes was measured, and their content was calculated using equation I.

#### 2.4.2. Uronic acid determination

The uronic acid content was analyzed using GalA as the standard by the m-hydroxy biphenyl method (28–30). The absorbance of prepared analytes was determined under the wavelength of 525 nm to build standard equation II, *y* = 6.7717 × + 0.1089, *R*^2^ = 0.9996. The PKP2-1 polysaccharide solutions were measured, and their contents were calculated based on equation II.

#### 2.4.3. Homogeneity analysis

The UV–vis spectrum of PKP2-1 was collected using a SHIMADZU UV-2550 version spectrophotometer in full-wavelength scanning mode ranging from 200 to 800 nm. The molecular weight of PKP2-1 was detected using GPC on an HPLC system (Agilent 1260 series) loaded with a refractive index detector (RID). Briefly, PKP2-1 (1 mg/mL) was delivered to an SRT SEC-150 column (7.8 × 300 mm) and eluted using ultra-pure water at a flow rate of 0.8 mL/min. The molecular weight of PKP2-1 was estimated based on a calibration curve plotted with a series of dextran standards (1, 5, 12, 50, and 150 kDa).

#### 2.4.4. FT-IR spectroscopy

PKP2-1 (2 mg) was mixed with dried potassium bromide (200 mg) to ground and pressed in a vacuum. The IR spectrum of PKP2-1 was acquired using a Nicolet 5700 FT-IR spectrometer in the wavelength region of 4,000–500 cm^–1^.

#### 2.4.5. Monosaccharide composition

The monosaccharide composition was analyzed using the Gas chromatography-single quadrupole mass spectrometry (GC-MS) method (31).

Briefly, PKP2-1 (20 mg) was added in 2 mL of TFA with a concentration of 2 mol/L, hydrolyzed at 100°C for 6 h, and the solvent was evaporated under reduced pressure. The dried acid-hydrolyzed analyte was dissolved in anhydrous DMSO (2 mL) and NaOH power (60 mg), stirring at 35°C for 30 min, and then 1 mL of CH_3_I was slowly added. The reaction mixture was sealed up and continued to react in the dark for 12 h, then distilled water (2 mL) was added to stop it. The methylated sample was extracted using dichloromethane and analyzed by the GC-MS method. The monosaccharide standards, including Rha, Ara, Fru, Gal, Glc, Xyl, Man, GalA, and GlcA, were also analyzed using the above method.

#### 2.4.6. Methylation assay

Glycosidic linkage analysis for PKP2-1 was implemented by referencing a previously reported method with a little modification (32). The uronic acid was first reduced to neutral sugar before the methylation analysis.

In brief, 10 mg of dried PKP2-1 was completely dissolved in anhydrous DMSO (2 mL). After adding NaOH (60 mg), the reaction solution was stirred at 35°C for 2 h. Subsequently, 1 mL CH3I was slowly added, and the reaction mixture was placed in the dark for 12 h. After adding 2 mL of ultra-pure water, the methylated sample was extracted using dichloromethane and washed thrice using ultra-pure water. Complete methylation of the PKP2-1 was confirmed by observing the peak disappearance at 3,200–3,700 cm^–1^ wavelength in the infrared spectrum. The complete methylated product was further hydrolyzed using 2 mL of 2 M TFA at 100°C for 6 h. After removing excess TFA, NaBH_4_ (30 mg) and 0.05 M NaOH solution (1 mL) were successively added. After 12 h, 100 μL of acetic acid was added, and the solvent was removed in a vacuum. Subsequently, pyridine and acetic anhydride (each 1 mL) were added, sealed and stirred for 2 h at 90°C. The reacted solution was extracted using dichloromethane and detected through the aforementioned GC-MS method.

#### 2.4.7. 1D and 2D NMR analysis

Dried polysaccharide PKP2-1 (20 mg) was dissolved into 0.6 mL of D_2_O to achieve the NMR measurements. The 1D ^1^H NMR and ^13^C NMR, and 2D ^1^H-^1^H COSY, HSQC, HMBC, and NOESY spectrum of the PKP2-1 were recorded using an NMR spectrometer with 600 Hz frequency at 60°C. The ppm was used as a unit of chemical shifts.

### 2.5. Anti-inflammatory activities *in vitro*

#### 2.5.1. Cell culture

The assays were split into five groups: the normal group, model group, low-dose treated group, medium-dose treated group, and high-dose treated group. Human MH7A cells were maintained in DMEM supplemented with 10% fetal bovine serum (FBS), 100 U/mL penicillin and 100 μg/mL streptomycin under a humidified 5% carbon dioxide condition. Briefly, MH7A cells were seeded into a 96-well plate (1 × 10^6^/well) and cultured at 37°C. After the cells completely adhered to the wall, they were induced with TNF-α (20 ng/mL) for 0.5 h and incubated using PKP2-1 solution at a concentration of 50 μg/mL (low-dose), 100 μg/mL (medium-dose), and 200 μg/mL (high-dose), respectively, for 24 h. After the treatment using PKP2-1, the cells were washed thrice with PBS solution and incubated again in DMEM (33, 34).

#### 2.5.2. Cell viability and ELISA assays

Cell viability was assessed using a cell counting kit-8 (CCK-8) assay. Following routine cell culture, an additional process was added. After 24 h of incubation, 10 μL of CCK-8 reagent was added to each well and incubated at 37°C for another 1 h. Then, a microplate reader was used to determine its absorbance at 450 nm. In addition, the cell supernatants were collected, and the content of IL-1β, IL-6, and IL-10 was determined using commercial ELISA kits.

#### 2.5.3. DAPI staining

To determine whether the anti-proliferative effects of PKP2-1 on MH7A cells could induce apoptosis, DAPI staining analysis was performed. Following routine cell culture, an additional process was added. After treatment with PKP2-1, the cells were washed thrice with PBS and fixed with 4% paraformaldehyde for 15 min at room temperature. Then, the cells were stained with a DAPI solution in the dark for 10 min, followed by washing with PBS and photographed under an Olympus CKX53 inverted fluorescence microscopy.

#### 2.5.4. Apoptosis

Flow cytometry analysis stained with FITC conjugated Annexin V/PI was conducted to determine the apoptosis-inducing effects of PKP2-1 in MH7A cells. Briefly, MH7A cells were seeded into a 6-well plate (1 × 10^6^/well) and cultured at 37°C. When the cells completely adhered to the wall, they were induced with TNF-α (20 ng/ml) for 0.5 h, then incubated with different concentrations of PKP2-1 (50, 100, and 200 μg/mL, respectively) for 24 h. Subsequently, the reaction mixture was digested by trypsin-EDTA, washed twice using cold PBS solution, and centrifuged at 1,500 rpm for 2 min to collect supernatant cells. Next, the supernatant cells were suspended by successively adding 500 μL 1 × Binding Buffer and 5 μL of Annexin V-FITC. After 15 min in the dark, 5 μL of propidium iodide was added, and the final cell solution was incubated at 37°C for another 5 min.

#### 2.5.5. Cell cycle

Next, we performed a cell cycle assay. Following routine cell culture, an additional process was added. The supernatant was discarded, and attached cells were collected. Subsequently, the cells were maintained at −20°C for 24 h. After staining with propidium iodide, the DNA content was determined by FACS Calibur flow cytometry.

#### 2.5.6. Scratching assays

Cell migration assay was performed by the scratch wound healing method. MH7A cells (1 × 10^6^/well) were seeded into a 6-well plate. When the cells covered the whole bottom of the well, the serum-free medium was applied to continue the culture for 12 h. Next, a scratch at the bottom of the well was established using a pipet tip, and the cells were induced using TNF-α (20 ng/mL) for 0.5 h and treated using PKP2-1 (50, 100, and 200 μg/mL). After 24 h of scratching, the cells were stained, and an inverted microscope was applied to observe and photograph the scratch area.

#### 2.5.7. Transwell experiments

The Transwell chamber was exploited to assess cell migration and invasion capacity. Briefly, 0.1% gelatin was coated on the transwell for 30 min at 37°C and washed using PBS solution thrice. The cells (1 × 10^6^/well) were seeded into the upper chambers of the transwell and induced with TNF-α (20 ng/mL) for 0.5 h, then incubated using PKP2-1 (50, 100, and 200 μg/mL) for 24 h, whereas the bottom chambers were filled with the DMEM medium containing 20% FBS. Notably, cells at the bottom side of the membrane were collected as invasive cells and fixed with 4% paraformaldehyde. The invasive cells were washed with PBS three times, and then stained with crystal violet. Lastly, an optical microscope was used to observe the invasive cells. The Image J software was used to count the change in invasive cells.

### 2.6. Statistical analysis

Graphs were made using GraphPad Prism 7.0, and data were analyzed using the SPASS 22.0 software. Statistical evaluations in all experiments were performed using one-way ANOVA, followed by a student’s *t*-test. All experiments were repeated in triplicate, and all data are presented as mean ± standard deviation (SD). Statistical significance was set at p < 0.05.

## 3. Results

### 3.1. Homogeneity analysis

**FIGURE 1:**
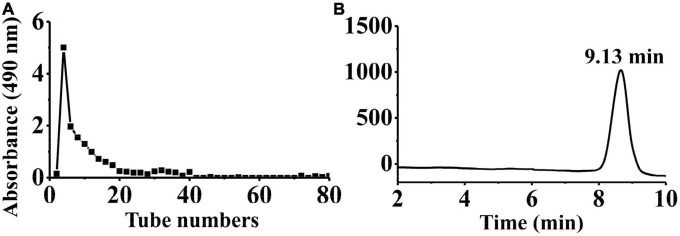
Physicochemical property analysis of PKP2-1. **(A)** The SEC profiles for PKP2 elution; **(B)** The HPGPC analysis for PKP2-1.

Crude PKP (64.88 g) was obtained from the rhizomes of P. *kingianum* Coll. and Hemsl (1,300 g). Then, 800 mg of crude PKP was eluted using 0.05 M NaCl solution at a flow rate of 2.0 mL/min to yield PKP2 (148 mg, Supplementary Figure 1A). It was further fractionated through GPC to obtain the target polysaccharide, PKP2-1 (72.8 mg), as shown in Figure 1A. The total sugar and uronic acid content of PKP2-1 were 84.22% (w/w) and 6.92% (w/w), respectively. No absorption in the UV spectra at 260 and 280 nm revealed that the ingredient of PKP2-1 was free of nucleic acids and protein, respectively (Supplementary Figure 1B) (35). The standard curve of molecular weight was LogMw = −0.44 × + 8.26, *R*^2^ = 0.9988. The retention time of PKP2-1 was 9.13 min in the HPGPC, and thus its molecular weight was calculated as 17.34 kDa based on the retention time (Figure 1B).

### 3.2. FT-IR analysis

**FIGURE 2:**
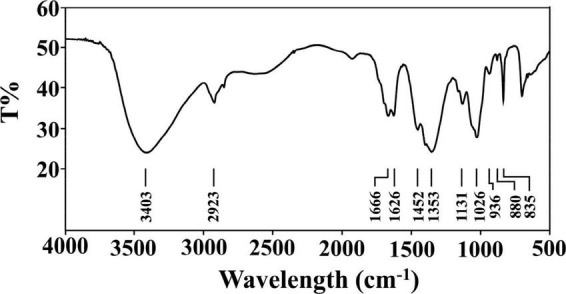
FT-IR spectrum of PKP2-1 in the frequency range of 500–4,000 cm^– 1^.

The IR spectrum of PKP2-1 is shown in Figure 2. First, PKP2-1 did not contain a protein because of the absence of absorption peaks at 1,541 cm^–1^ (36). Second, the wide peak at 3,403 cm^–1^ was responsible for the presence of the –OH group in the PKP2-1 (37). The peak at 2,923 cm^–1^ was the C-H asymmetric stretching vibration from the CH_3_ group. The two peaks at 1,666 cm^–1^ and 1,452 cm^–1^ were the –COOH bending vibration, which further confirmed that uronic acid occurred in polysaccharide molecules (38). Absorption peaks at 1,131 and 1,026 cm^–1^ indicated the asymmetric stretching vibration of C-O-C. These results demonstrated that PKP2-1 was a type of polysaccharide.

### 3.3. Monosaccharide compositions and glycosidic linkages

The monosaccharide compositions of PKP2-1 were determined by GC-MS analysis after TFA hydrolysis, followed by derivatization. The results revealed that PKP2-1 consisted of Glc, Gal, Man, and GlcA in an approximate molar ratio of 6:2:2:1 (Supplementary Figure 2). The methylation and acetylation reactions were further conducted to identify the connection type of glycosidic linkages in PKP2-1 (39). Combined with the published literature (40), methylation analysis indicated that PKP2-1 consisted of (→2)Glcp(4→, Glcp(3→, →2)Manp(3→, →2,3)Galp(4→, →1)Galp(4→ at the molar ratio of 27.37:23.53:29.87:2.85:16.38). The corresponding results are listed in Table 1.

**TABLE 1:** GC–MS analysis for methylation experiments of PKP2-1.

Methylation product	TR (min)	Linkage type	Main MS (m/z)	Molar ratio (%)
3,5-di-O-acetyl-2,4,6-tri-O-methyl glucitol	6.81	Glcp(3→)	101, 88, 75, 59, 201	23.53
2,4,5-tri-O-acetyl-3,6-di-O-methyl glucitol	10.92	(→2)Glcp(4→)	75, 116, 87, 103, 127	27.37
2,3,5-tri-O-acetyl-4,6-di-O-methyl mannitol	13.18	(→2)Manp(3→)	71, 74, 87, 129, 102	29.87
1,4,5-tri-O-acetyl-2,3,6-tri-O-methyl galactitol	18.75	(→1)Galp(4→)	101, 75, 88, 161, 57	16.38
2,3,4,5-tetra-O-acetyl-1,6-tri-O-methyl galactitol	23.20	(→2,3)Galp(4→)	113, 87, 141, 129, 69	2.85

### 3.4. 1D and 2D NMR analysis

NMR spectroscopy is a common and significant measure for determining specific structural characteristics, such as confirming monosaccharide types, identifying α- or β-anomeric configurations and elucidating glycosidic linkages. Therefore, NMR analysis was performed to characterize the structure of PKP2-1 further. In terms of the data available from ^13^C and 1H spectra of PKP2-1, the various residue signals are detailed below.

#### 3.4.1. Preliminary analysis by NMR analysis

**FIGURE 3:**
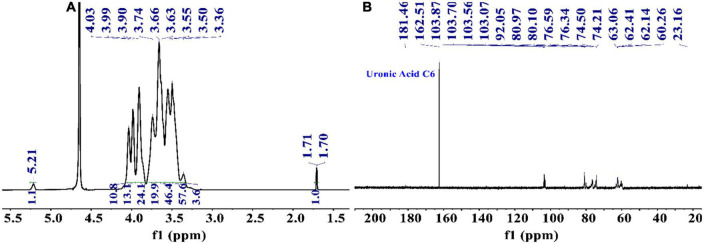
The structural elucidation of PKP2-1 by NMR analysis. **(A)** ^1^H NMR spectrum; **(B)** ^13^C NMR spectrum.

From the ^1^H NMR spectrum shown in Figure 3A, the appearance of δ5.21 ppm signal indicated a residue of α-configuration occurred in the structure of PKP2-1, which was assigned to H1 of residue A (41, 42). Moreover, the region (4.03–3.36 ppm) was responsible for H2-H6 of residues (A–E) presented in PKP2-1. In ^13^C NMR spectra depicted in Figure 3B, the five peaks at 103.87, 103.70, 103.56, 103.07, and 92.05 ppm were attributable to the anomeric signals located on C1 of residues A, B, C, D, and E, respectively (43). In the higher-field region, the δ181.46 and δ162.51 ppm carbon signals were responsible for the carbonyl of uronic acid and ester group, respectively (44).

**FIGURE 4:**
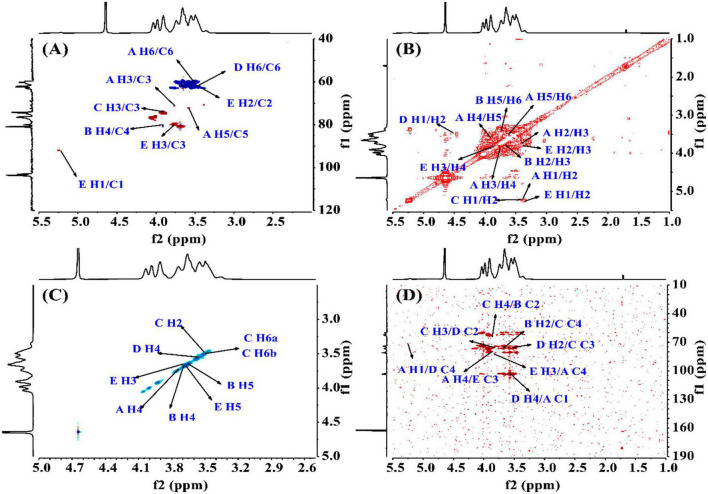
The structural elucidation of PKP2-1 by NMR analysis. **(A)** HSQC NMR spectrum; **(B)** ^1^H-^1^H COSY NMR spectrum; **(C)** NOESY spectrum; **(D)** HMBC spectrum.

To find the relation between the anomeric carbon signals and corresponding protons, the HSQC spectrum shown in Figure 4A was investigated. The results revealed that the signals at δ5.24/92.05 ppm were responsible for heterologous region H1/C1 of the residue E. The ^1^H-^1^H COSY (Figure 4B) and NOESY (Figure 4C) spectrum showed five cross peaks at δ5.24/3.38, 3.38/3.74, 3.74/3.88, 3.88/3.59, and 3.59/3.51 ppm which were consistent with the H2-H6 signal of residue E at 3.38, 3.74, 3.88, 3.59 and 3.51 ppm (45).

#### 3.4.2. Elucidating glycosidic linkages

The HMBC spectrum showed the correlation analysis among five residues (A–E). As shown in Figure 4D, the H2 signal of residue B (3.65 ppm) and the C4 of residue C (76.22 ppm), and the H4 signal of residue C (3.85 ppm) and the C2 of residue B (79.97 ppm) presented mutual connection, indicating the existence of 2,4-linkage between residue B and C. Additionally, the C1 signal of residue A (103.87 ppm) and the H4 of residue D (3.57 ppm), and the C4 signal of residue D (80.91 ppm) and the H1 of residue A (5.22 ppm) showed a positive interaction, which was responsible for the 1,4-linkages type between residue A and D. The existence of 2,3-linkage type between residue C and D was also observed from the HMBC spectrum. The H2 signal of residue D (3.50 ppm) and the C3 of residue C (76.34 ppm), and the C2 signal of residue D (81.90 ppm) and the H3 of residue C (3.82 ppm) were highly correlated. Lastly, the C3 signal of residue E (79.90 ppm) and the H4 of residue A (3.88 ppm), and the H3 signal of residue E (3.77 ppm) and the C4 of residue A (80.97 ppm) demonstrated the 3,4-linkages type between residue E and A. The ^13^C and ^1^H NMR spectra of residues A-E were analyzed based on 1D ^1^H, ^13^C NMR, 2D COSY, HSQC, HMBC, and NOESY. The complete assignment of PKP2-1 is shown in Table 2.

**TABLE 2:** Chemical shifts of resonances in the ^1^H NMR and ^13^C NMR spectra of PKP2-1.

No.	Glycosyl residues	H1/C1	H2/C2	H3/C3	H4/C4	H5/C5	H6/C6
A	(→1)-α-D-Galp(4→)	5.22/103.87	3.38/70.76	3.74/71.43	3.88/80.97	3.59/76.33	3.50/60.26
B	(→2)-α-D-Manp(3→)	–/103.70	3.65/79.97	3.78/80.10	3.90/70.76	3.68/79.07	3.30/62.14
C	(→2,3)-α-D-Galp(4→)	5.21/103.56	3.47/82.56	3.82/76.34	3.85/76.22	3.60/74.50	3.50/61.95
D	(→2)-β-D-Glcp(4→)	4.46/103.07	3.50/81.90	3.82/74.34	3.57/80.90	3.64/74.21	3.46/62.41
E	α-D-Glcp(3→)	5.24/92.05	3.37/62.27	3.77/79.90	3.86/74.34	3.72/76.59	3.44/63.06

### 3.5. Structural deduction

**FIGURE 5:**
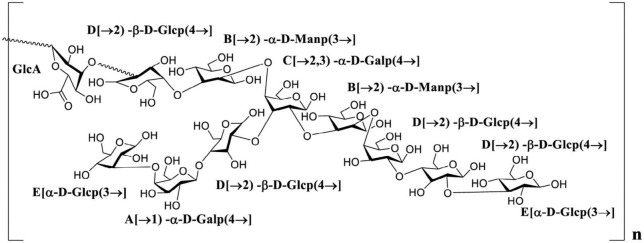
The putative structure of PKP2-1.

Polysaccharides extracted from natural sources have diverse crucial biological functions directly influencing life processes. The physicochemical properties and structural characteristics, including total sugar content, monosaccharide composition and glycosidic linkage, chain conformation and degree of branching, directly affected the biological activity of polysaccharides. Combined with the result of monosaccharide components, glycosidic linkages, NMR analysis and previous reports, the PKP2-1 structure was presumably identified as a heteroglycan consisting of (→2, 3)-α-D-Galp(4→, →2)-α-D-Manp(3→, →2)-β-D-Glcp(4→ and α-D-Glcp(3→ residues with side chains →2)-β-D-Glcp(4→, →1)-α-D-Galp(4→) and α-D-Glcp(3→ branches located at O-3 position of →2, 3)-α-D-Galp(4→, and GlcA may be located at O-2 of →2)-β-D-Glcp(4→), and its structural unit is presented in Figure 5.

### 3.6. Anti-inflammatory activity *in vitro*

There was a close relationship between inflammatory reaction and radical oxidation. In this study, we carefully evaluated the antioxidant activity of PKP1 and PKP2 to determine the higher activity portion for further anti-inflammatory assay. Antioxidant assays included scavenging DPPH radical, hydroxyl radical and reducing power (Fe^3+^ to Fe^2+^) (46–48). The results demonstrated that the PKP2 portion exhibited higher radical-scavenging abilities, as presented in Supplementary Figure 3. Subsequently, the PKP2 portion was further purified by the Sephadex G-200 column to obtain PKP2-1. The PKP1, PKP2 and PKP2-1 showed effective antioxidant activity. Briefly, the radical scavenging rate of three analysts in the range of 2–10 mg/mL was in the following order: PKP2-1 > PKP2 > PKP1, indicating that PKP2-1 had a stronger antioxidant performance. Thus, PKP2-1 was selected to research further.

#### 3.6.1. Cell proliferation

**FIGURE 6:**
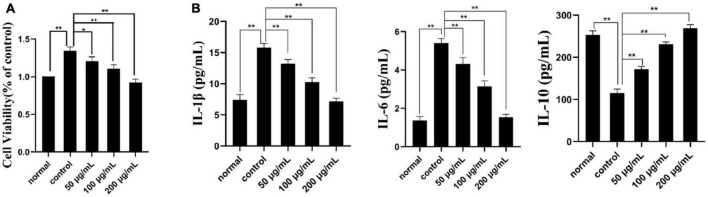
Cell viability and ELISA assays. **(A)** The effect of PKP2-1 on the proliferation of MH7A cells; **(B)** The effect of PKP2-1 on the release of IL-6, IL-10, and IL-1β from MH7A cells stimulated by TNF-α. *p < 0.05, **p < 0.01 compared to blank control group.

CCK-8 assay is a reliable method to detect cell proliferation or survival rate (49). As shown in Figure 6A, the concentration of the normal group, model group and three PKP2-1 groups were 50, 100, and 200 μg/mL and were treated with MH7A cells for 24 h. The cell viability of the normal group was set as 100%. When TNF-α (20 ng/mL) stimulated the MH7A cells for 1 h, corresponding cell viability was calculated as 135%, implying that inflammation was promoted. Notably, treatment with MH7A cells using the PKP2-1 at a concentration of 50, 100, and 200 μg/mL for 24 h showed that the cell viabilities were 120, 110, and 91%, respectively. Therefore, the survival rate of MH7A cells decreased while the concentration of PKP2-1 increased.

#### 3.6.2. ELISA determination

The effects of PKP2-1 on the expression of cytokines IL-1β, IL-6, and IL-10 in MH7A cells was measured by the ELISA method, and corresponding results are shown in Figure 6B. In the normal group, the content of pro-inflammatory cytokines IL-6 and IL-1β in MH7A cells were 1.36 and 7.41 pg/mL, respectively. Following stimulation with TNF-α (20 ng/mL), the content of IL-6 and IL-1β have transferred to 5.40 and 15.81 pg/mL, respectively, which was significantly higher than those in the normal group. However, following treatment with MH7A cells using the PKP2-1 at a concentration of 50, 100, and 200 μg/mL for 24 h, we observed that the content of IL-6 and IL-1β decreased (the content of IL-6 and IL-1β was 4.3, 3.14, 1.54 pg/mL and 13.22, 10.24, 7.15 pg/mL, respectively.), suggesting that PKP2-1 could inhibit the production of pro-inflammatory cytokines. Meanwhile, the content of anti-inflammatory cytokine IL-10 in the normal group and model group (stimulating with 20 ng/mL TNF-α) were 253.41 and 115.32 pg/mL, respectively. When the MH7A cells were treated with PKP2-1 at a concentration of 50, 100, and 200 μg/mL for 24 h, the content of IL-10 was 171.65, 231.01 and 269.18 pg/mL, respectively, implying that PKP2-1 could promote the production of anti-inflammatory cytokines. These results indicated that PKP2-1 possessed anti-inflammatory activity in a dose-dependent manner.

#### 3.6.3. Mechanism investigation by MH7A cells

To explore the potential anti-inflammatory mechanisms of PKP2-1 in MH7A cells, the apoptosis-inducing assay of PKP2-1 was assessed following DAPI staining and flow cytometry analysis (33, 50).

##### 3.6.3.1. DAPI staining

**FIGURE 7:**
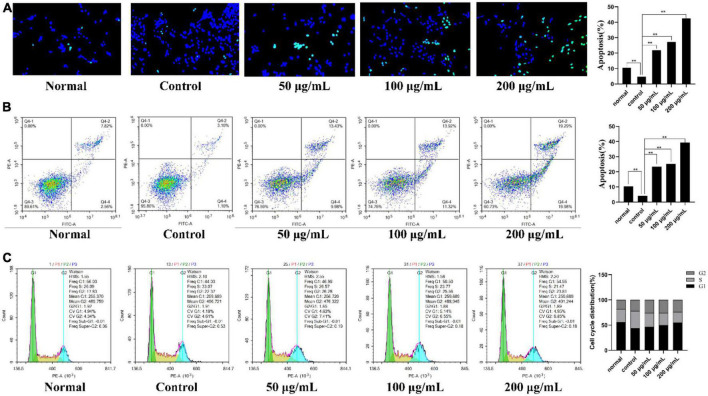
Mechanism investigation of PKP2-1 in anti-inflammatory activity based on MH7A cells. **(A)** Apoptosis-inducing effect of PKP2-1 on MH7A cells; **(B)** Apoptotic assay by flow cytometry assay; **(C)** The effect of PKP2-1 on the cell cycle of MH7A cells; MH7A cells were treated with TNF-α (20 ng/mL) and PKP2-1 (50, 100, and 200 μg/mL) for 24 h, and apoptotic cells were detected by Annexin V-FITC\/PI staining, followed by flow cytometry Cytological analysis, Data were represented as Mean ± SD (*n* = 3), **p < 0.01, vs. Control cells (MH7A cells treated by TNF-α alone).

As shown in Figure 7A, intact cell nuclei demonstrated faint DAPI fluorescence in the normal, and TNF-α stimulated MH7A cells. In comparison, MH7A cells treated with PKP2-1 (50, 100, and 200 μg/mL, respectively) showed typical nuclear morphological changes of apoptosis, such as nuclear condensation, increased brightness and nuclear crinkle. Further analysis using the flow cytometry technique and Annexin V-FITC/PI staining confirmed the pro-apoptotic effects of PKP2-1. A gradual increase in the apoptosis rate could be observed in Figure 7B, suggesting that PKP2-1 could induce apoptosis of synovial fibroblasts, thus achieving positive pro-apoptotic and anti-inflammatory effects.

##### 3.6.3.2. Apoptosis and cell cycle assay

After reaction with different concentrations of PKP2-1 for 24 h, the cell cycle distribution of MH7A cells was measured by flow cytometry, and the cell cycle fitting curve was profiled by the FlowJo software. Cell cycle refers to the process whereby cells proliferate by mitosis and divide into interphase and division (M phase). The interphase is further divided into the G1, S, G2, and G0 phases. As shown in Figure 7C, the cell number ratio of the model group in the G0/G1 phase was 44.03%. After treatment using 50 μg/mL of PKP2-1 for 24 h, the ratio reached 46.96%. Further, the G0/G1 phase ratio with PKP2-1 concentration of 100 and 200 μg/mL could reach 50.50 and 54.55%, respectively. Moreover, the ratio of cell number in the S phase decreased from 33.07% in the control group to 26.57% in 50 μg/mL of the PKP2-1 group and further reduced to 23.77 and 21.47% with 100 and 200 μg/mL of PKP2-1 group, respectively. The results displayed that the number of cells in the G0/G1 phase of MH7A cells gradually increased with the increase of PKP2-1 concentration, while the number of cells in the S phase gradually decreased. The cycle was blocked in the G0/G1 phase.

##### 3.6.3.3. Scratching assays

**FIGURE 8:**
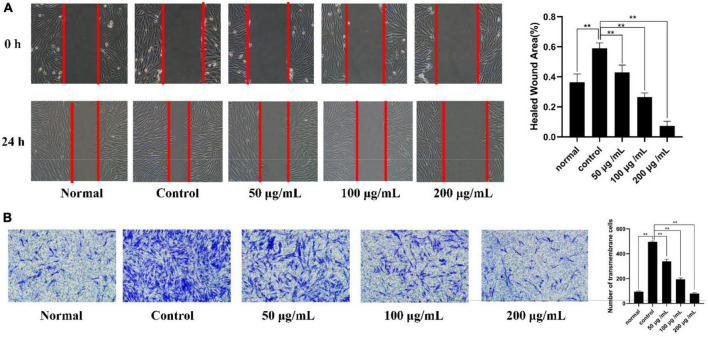
Mechanism investigation of PKP2-1 in anti-inflammatory activity based on MH7A cells. **(A)** Statistical results of the effect of PKP2-1 on the migration of MH7A cells; **(B)** Transwell chamber detection of the effect of PKP2-1 on the migration of MH7A cells. MH7A cells were treated with TNF-α (20 ng/mL) and PKP2-1 (50, 100, and 200 μg/mL) for 24 h, Data were represented as Mean ± SD (*n* = 3), **p < 0.01, vs. Control cells (MH7A cells treated by TNF-α alone).

The migration ability of the cells was evaluated with the cell scratch assay by measuring the scratch width. First, the healing state of MH7A cells in the normal group, control group and different concentrations of PKP2-1 (50, 100, and 200 μg/mL) groups were observed under an inverted microscope at 0 h. After 24 h, the scratch width was observed and recorded (Figure 8A), and the rate of cell migration was calculated. The results showed that the rates of cell migration in the normal group and model group were 36.4 and 59.0%, respectively. Comparatively, the rates of MH7A cell migration after 24 h following 50, 100, and 200 μg/mL of PKP2-1 treatment were 43, 26, and 7%, respectively. The migration rate of MH7A cells decreased in the 50 μg/mL PKP2-1 group and significantly reduced when the PKP2-1 concentration reached 200 μg/mL, indicating that PKP2-1 could inhibit the migration of MH7A cells.

##### 3.6.3.4. Transwell experiments

Additionally, Transwell experiment was performed to determine further the anti-invasive effect of PKP2-1 on TNF-α-stimulating MH7A cells (Figure 8B). The results showed that when PKP2-1 acted on MH7A cells, the average number of transmembrane migrations decreased. When the MH7A cells passed through the inner membrane of the chamber to the outer environment, the number of cells was significantly reduced in three PKP2-1 groups compared with the control group (*p* < 0.05). These results suggested that PKP2-1 could inhibit the invasion of MH7A cells.

#### 3.6.4. Mechanism investigation by RAW264.7 cells

##### 3.6.4.1. Cell viability

Supplementary Figure 4A shows that the proliferation experiment of RAW 264.7 cells showed 100% viability for the normal and 133% for the model. This indicates that the model was successful. A positive control group treated with MTX also had a survival rate of 107%. RAW 264.7 cells were treated with different concentrations of compound PKP2-1 for 24 h. A higher concentration of compound PKP2-1 led to a higher survival rate for RAW 264.7 cells than in the blank group. At concentrations of 50, 100, and 200 μg/mL intervened in RAW 264.7 cells for 24 h, the corresponding cell viability was 122, 113, and 94%, respectively.

##### 3.6.4.2. Cytokine factors

A comparison of the effects of different concentrations of PKP2-1 on NO secretion by macrophages induced by LPS is shown in Supplementary Figure 4B. Compared to the blank control group, LPS-induced macrophages produced significantly more NO, indicating a successful establishment of an inflammation model induced by LPS. In comparison to the LPS group, AP1-b treatment significantly reduced the production of NO in the medium between 50 and 200 g/mL. In the positive control group, the PKP2-1 treatment produced 5.98 mol/L of NO, whereas 200 g/mL of PKP2-1 produced only 5.98 mol/L of NO, 62% lower than that of the LPS treatment. According to the results, PKP2-1 inhibited NO release by macrophages induced by LPS, suggesting that it has anti-inflammatory properties. Furthermore, we also observed the effect of PKP2-1 on the release of TNF-α and IL-β in RAW 264.7 cells after LPS-stimulated. TNF-α and IL-β in RAW 264.7 cells after LPS stimulation significantly increased (compared with the normal group, *p* < 0.01), but PKP2-1 (50, 100, and 200 mg/mL) significantly decreased the release of TNF-α and IL-6 in RAW 264.7 cells after LPS-stimulated (compared with the control group, *p* < 0.01).

## 4. Discussion

### 4.1. Monosaccharide compositions in *P. kingianum*

Traditional Chinese medicine polysaccharides play a crucial part in the prevention and treatment of diseases. Their physicochemical properties and structural characteristics significantly affected their biological activity. Previous reports that PKPs were isolated from *P. kingianum*. (13, 21, 51–54) were listed in Table 3. With our interest in exploring TCMs related to anti-inflammatory (55–58), in particular *Polygonatum* species (22, 23), we investigated the correlation between anti-inflammatory activity and the structure of *Polygonatum* polysaccharides. PKP possesses versatile bioactivity, including antioxidant activity, antidiabetic activity, antihyperlipidemic effect, etc., Glc and Gal are the main monosaccharide compositions.

**TABLE 3:** The polysaccharides isolated from *Polygonatum kingianum*.

No.	Name	Monosaccharide composition	Molecular weight	Structures	Bioactivity	References
1	PS	Man Gal, Glc, Ara, GalA, Rib, and Rha	134.7 kDa	/	Improving intestinal probiotics	(51)
2	PS	Man, GalA, Gal, and Fru	178.6 kD	/	Antihyperlipidemic effect	(52)
3	PKP	Gal, GalA, Ara and Glc	/	/	Antidote	(13)
4	PKP	Gal, Glc, Fru, GalA	8.7 kDa	/	Antioxidant activity	(53)
5	PKP	Glc, Man, GalA, Gal, GlcA, and Ara	14.05 kDa	(1→4) linked glc	Antidiabetic activity	(21)
6	PSP	Man, GalA, GlcA, Gal, Ara and Fru	85.02 kDa	/	Antioxidant activity	(54)

### 4.2. Glycosidic linkage in PKPs

Although polysaccharides have various biological activities, molecular structure analysis of PKP mostly focuses on monosaccharide composition and molecular weight determination. There has been a lack of investigations on the glycosidic linkage and NMR. 1,4-β-D-Glcp was considered the main glycosidic linkage in *Polygonatum* species. However, present studies showed that 2,4-β-D-Glcp and terminal α-D-Glcp(3→) are the main structural unit.

### 4.3. Activity evaluation of PKPs

In this present study, we found that PKP2-1 was a novel polysaccharide and showed strong activity in radical scavenging and anti-inflammatory effects on MH7A inflammatory cells in a concentration-dependent manner. We speculate that the anomeric configurations and monosaccharide composition and content jointly determined the unique anti-inflammatory activity of PKP2-1. On the basis of monosaccharide compositions and glycosidic linkage analysis, the researchers found Glc, Man and Gal are commonly major constituents, and 1,2-link glucose or 1,4-link mannose occurs in the backbone of PKP (13, 21, 53). In the present study, PKP2-1 was characterized by a high level of Glc, 27.37% of (→2)-β-D-Glcp-(4→), and molecular weight of approximately 17.34 kDa, which might be the reason for strong radical scavenging and anti-inflammatory activity.

## 5. Conclusion

A novel homogeneous polysaccharide, PKP2-1, was successfully obtained from *Polygonatum kingianum* by integrating several methods. The structure of PKP2-1 was elucidated *via* monosaccharide composition analysis, methylation experiments and 2D NMR investigations. The backbone of PKP 2-1 consisted of (→2, 3)-α-D-Galp(4→, →2)-α-D-Manp(3→, →2)-β-D-Glcp(4→) and α-D-Glcp(3→) residues with side chains →2)-β-D-Glcp(4→, →1)-α-D-Galp(4→ and α-D-Glcp(3→) branches located at O-3 position of (→2, 3)-α-D-Galp(4→). GlcA could be located at O-2 of (→2)-β-D-Glcp(4→). Further anti-inflammatory activity evaluation demonstrated that PKP2-1 could inhibit the growth of MH7A cells in a concentration- and time-dependent manner. Mechanistic studies showed that PKP2-1 could reduce the expression of IL-1β and IL-6 and promote the level of IL-10. Moreover, PKP2-1 induced apoptosis of synovial fibroblasts and inhibited the migration and invasion of MH7A cells. Thus, these results highlighted that PKP2-1 might be a potential antiphlogistic drug and immunomodulator. A detailed study of the anti-inflammatory mechanisms of PKP2-1 is ongoing in our research group.

## Data availability statement

The original contributions presented in this study are included in the article/Supplementary material, further inquiries can be directed to the corresponding authors.

## Author contributions

ZW: methodology, formal analysis, investigation, and writing–original draft. HL: methodology, formal analysis, and investigation. RF: methodology and formal analysis. JO: conceptualization and project administration. BW: project administration, writing–reviewing, and editing. All authors contributed to the article and approved the submitted version.
