# Supplementary material for: Structural characterization and anti-inflammatory activity of a novel polysaccharide PKP2-1 from Polygonatum kingianum
Source: Front Nutr. 2023 Mar 27;10:1156798. doi: 10.3389/fnut.2023.1156798 (PMC10083337; doi:10.3389/fnut.2023.1156798)
Supplement: Supplementary file 1 [file Data_Sheet_1.docx]

Supplementary Material

Structural characterization and anti-inflammatory activity of a novel polysaccharide PKP2-1 from *Polygonatum kingianum*

***Zhen Wang^1^†, Hui Liu^1^†, Ranze Fu^1^, Jinmei Ou^1*^ and Bin Wang^1*^***

^1^Key Laboratory of Xin'an Medicine of the Ministry of Education, Anhui University of Chinese Medicine, Hefei, China

**** Correspondence:*** *Jinmei Ou,* [*ojm@ahtcm.edu.cn*](mailto:ojm@ahtcm.edu.cn)；*Bin Wang,* [*bw5654@ahtcm.edu.cn*](mailto:bw5654@ahtcm.edu.cn)

# Supplementary Data

**1.1 Determination and analysis of PKP components by GC-MS**

**1.1.1 The chromatographic conditions**

The GC-MS instrument was equipped with a BR-17 column (0.25 μm × 250 μm × 30 m). Carrier gas: High purity helium (purity 99.999%), Nanjing Shangyuan Industrial Gas Factory; Column flow: 1.0 mL /min; Split injection was carried out, and the inlet temperature is 250 °C. The temperature program was set as follows: starting from 60 °C, at 20 °C /min to 100 °C, held for 2 min, then allowed to reach 180 °C at 4 °C/min. The MS conditions were EI ion source, electron energy 70 eV; Scanning range m/z 40-600; Standard library for mass spectrometry: NIST 11.L. Ion source, transmission line, and the four-stage bar temperature were 220, 280 and 40 °C, respectively.

**1.1.2 Preparation of reference solution (1)**

5 mg monosaccharide sample was dissolved in 1 mL anhydrous DMSO for 10 min by ultrasound. In the monosaccharide solution, 30 mg NaOH was added and stirred at 35 °C for 30 min. 0.5 mL CH_3_I was slowly added under 35 °C conditions for closed reaction in dark for 12 h, then 2 mL distilled water was added to stop the reaction. The methylated sample was extracted with 3 mL dichloromethane and washed with the same volume of distilled water, then the dried methylene chloride solution was diluted and detected by GC-MS. The internal standard was C_16_H_34_.

**1.1.3 Preparation of test sample solution**

Briefly, PKP2-1 (20 mg) was added in 2 mL of TFA with a concentration of 2 mol/L, hydrolyzed at 100 °C for 6 h, and the solvent was evaporated under reduced pressure. The dried acid-hydrolyzed analyte was dissolved in anhydrous DMSO (2 mL) and NaOH power (60 mg), stirring at 35 °C for 30 min, then 1 mL of CH_3_I was slowly added. The reaction mixture was sealed up and continued to react in the dark for 12 h, then distilled water (2 mL) was added to stop it. The methylated sample was extracted using dichloromethane and analyzed by the GC-MS method.

**1.2 In vitro antioxidant activities [2]**

### 1.2.1 DPPH free radical scavenging activity

The capacity of scavenging DPPH radicals with the help of PKP1 and PKP2 was detected according to a previous report with slight modifications. Briefly, first, fresh DPPH (0.2 mM in methanol, 2.0 mL), and PKP2-1 solutions (2.0 mL) at various concentrations (0, 1, 2, 3, 4 and 5 mg/mL) were prepared. Second, the reaction mixture of prepared DPPH and PKP2-1 solutions at various concentrations were mixed thoroughly and hatched at 25 ℃ for 30 min under the dark condition. The UV-2550 ultraviolet spectrophotometer recorded the absorbance of the mixture at 524 nm wavelength. Finally, the activity of DPPH radical scavenging was calculated according to the following equation:

DPPH radical scavenging rate（%）=$\left( 1-\frac{A_{sample}-A_{control}}{A_{blank}} \right)\times100\%$

where A_blank_ is the absorption of blank sample, A_sample_ is the absorption of the analytical sample, and A_control_ is the absorption of the background (replaced DPPH solution with distilled water).

### 1.2.2 Hydroxyl radical scavenging activity

The capacity of scavenging hydroxyl radicals with the help of PKP1 and PKP2 was detected according to a previous report.

Briefly, first, PKP2-1 solutions (1.0 mL) at various concentrations (0, 1, 2, 3, 4 and 5 mg/mL) were prepared. Second, the reaction mixture of prepared Sample solutions (1 mL) at various concentrations (0, 1, 2, 3, 4 and 5 mg/mL), 1 mL of FeSO_4_ solution (6 mM), 1 mL of H_2_O_2_ (6 mM), and 1 mL of salicylic acid (2 mM in ethanol) were mixed thoroughly and hatched at 37 °C for 30 min. The UV-2550 ultraviolet spectrophotometer recorded the absorbance of the mixture at a wavelength of 510 nm. Finally, the activity of hydroxyl radical scavenging was calculated according to the following equation:

Hydroxyl radical scavenging rate（%）=$\left( 1-\frac{A_{sample}-A_{control}}{A_{blank}} \right)\times100\%$

where A_blank_ is the absorption of blank sample, A_sample_ is the absorption of the analytical sample, and A_control_ is the absorption of the background (replaced H_2_O_2_ solution with distilled water).

### 1.2.3 Reducing power assay

The reducing power of PKP1 and PKP2 was detected according to a previous method. Briefly, first, PKP2-1 solutions (1.0 mL) at various concentrations (0, 1, 2, 3, 4 and 5 mg/mL) were prepared. Second, the reaction mixture of prepared Sample solutions (1 mL) at various concentrations (0, 1, 2, 3, 4 and 5 mg/mL), phosphate-buffered saline (1.6 mL, 0.2 M, pH = 6.6) and potassium ferricyanide K_3_[Fe (CN)_6_] (1.6 mL, 1.0% (w/v)) were mixed evenly and incubated at 50 °C for 20 min. Adding 1.6 mL trichloroacetic acid (10%, w/v) to the reaction, a desired supernatant was obtained by centrifugation at 5500 rpm for 15 min. A mixture of 2.0 mL of supernatant, 2.0 mL of distilled water and 0.5 mL of FeCl_3_ solution (0.1% (w/v)) was incubated at 25 ◦C for 10 min. Finally, the absorbance of the mixture was recorded at a wavelength of 700 nm by UV-2550 ultraviolet spectrophotometer.

These results presented in **Fig. S3.**

# References

1. Needs P, Selvendran R. Avoiding oxidative degradation during sodium hydroxide/methyl iodide-mediated carbohydrate methylation in dimethyl sulfoxide. Carbohydr. Res. 245(1) (1993) 1–10. [doi: 10.1016/0008-6215(93)80055-j](https://doi.org/10.1016/0008-6215(93)80055-j).

2. Chen H, Zeng J, Wang B, Cheng Z, Xu J, Gao W, Chen K. Structural characterization and antioxidant activities of Bletilla striata polysaccharide extracted by different methods, Carbohydr Polym 266 (2021) 118149. doi: 10.1016/j.carbpol.2021.118149.

# 2 Supplementary Figures

**
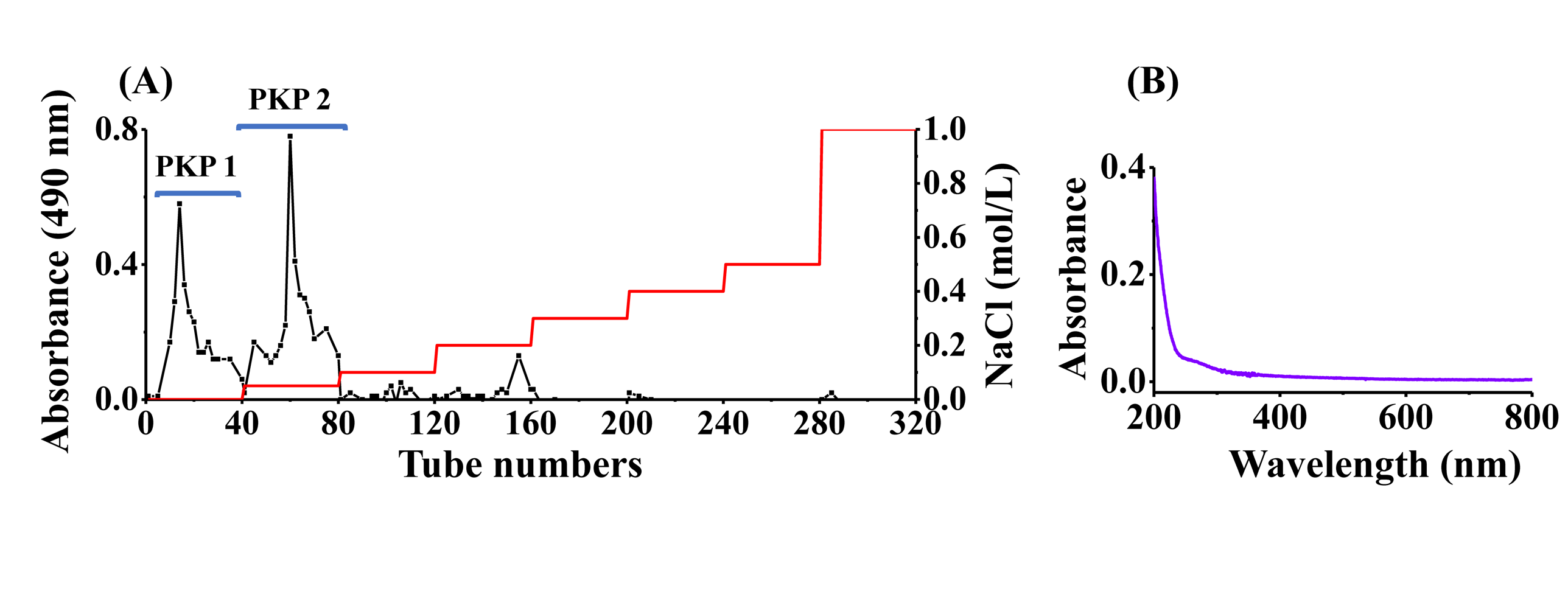
**

**Fig.S1** (A) Elution profiles of crude PKP by anion-exchange chromatography on a column of DEAE-cellulose. Two fractions were obtained, which were named PKP1 and PKP2. (B) The UV of PKP2-1.





**Fig. S2** Monosaccharide composition analysis for (A) Standard sample, (B)The PKP2-1 by GC method.


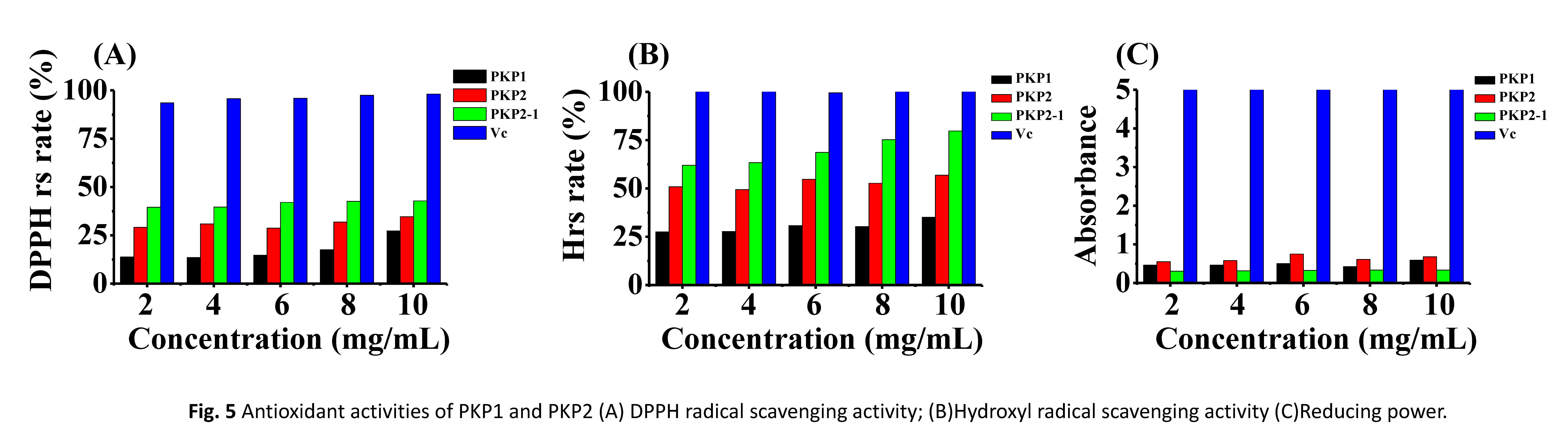


**Fig. S3** Antioxidant activities of PKP1, PKP2, PKP2-1 and Vc. Vc was used as the positive control. (A) DPPH radical scavenging activity; (B) Hydroxyl radical scavenging activity (C)Reducing power.


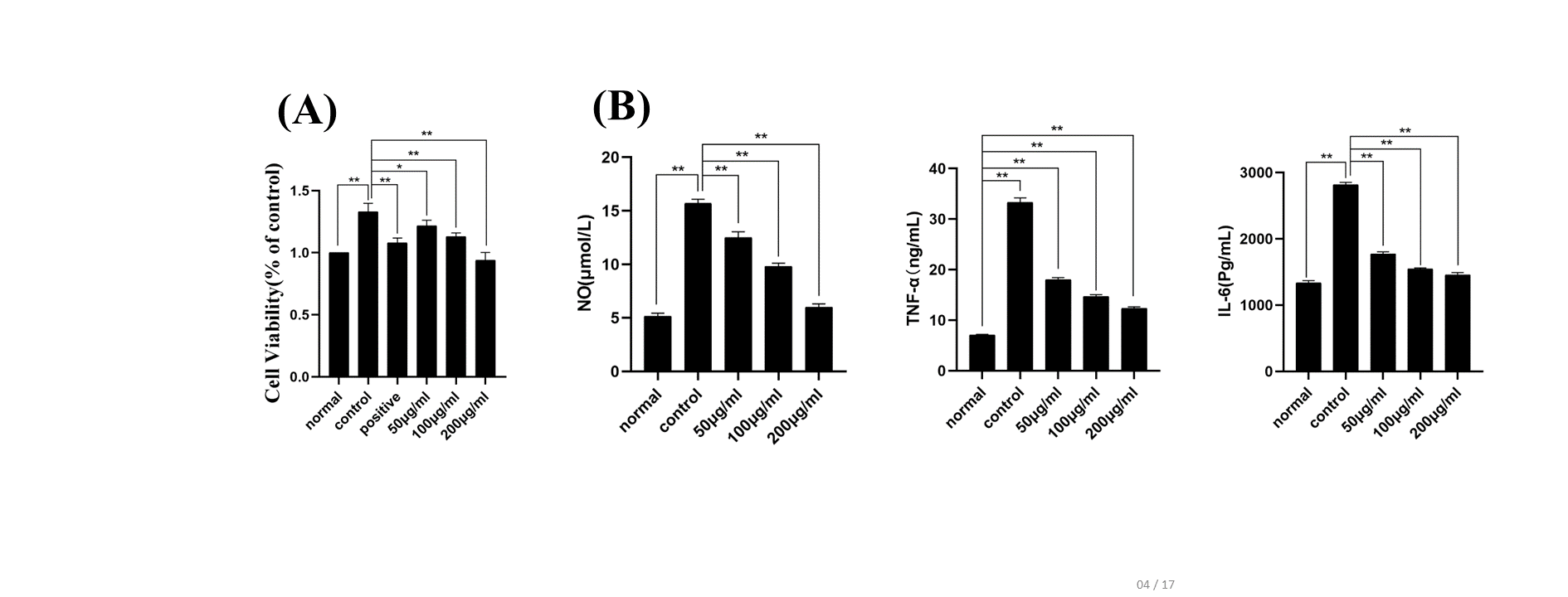


**Fig.S4** Cell viability and ELISA assays in RAW 264.7 cells. (A) The effect of PKP2-1 on the proliferation of RAW 264.7 cells; (B) The effect of PKP2-1 on the release of IL-6, IL-1β and NO from RAW.264.7 cells stimulated by TNF-α. *p < 0.05, **p < 0.01 compared to blank control group.
